# Virtual Electronic Tongue Combining Electrochemical Impedance Spectroscopy and the Artificial Neural Network for Accurate Identification of Noncompliant Gasoline

**DOI:** 10.1021/acsomega.5c08979

**Published:** 2026-02-27

**Authors:** Bianca de Paula Cola, André Guimarães de Oliveira, Ana Maria Rocco, Maiara Oliveira Salles

**Affiliations:** † Instituto de Química, Universidade Federal do Rio de Janeiro, Rio de Janeiro, 21941-909 RJ, Brazil; ‡ Conductive Materials and Energy Group, Postgraduate Program in Chemical and Biochemical Process Engineering, School of Chemistry, UFRJ, Universidade Federal do Rio de Janeiro, Centro de Tecnologia, Bloco E, Rio de Janeiro, 21941-909 RJ, Brazil

## Abstract

Noncompliant gasoline compromises engine performance, durability, and emissions. In this study, a virtual electronic tongue combining electrochemical impedance spectroscopy (EIS) and artificial neural networks (ANNs) was applied to identify and quantify common gasoline adulterants, namely, *n*-hexane, toluene, and mineral turpentine, in single- and multiadulterant systems. Measurements were performed using a glassy carbon electrode with platinum counter and pseudoreference electrodes. Single-adulterant systems exhibited increasing Nyquist semicircle diameters in the order *n*-hexane < mineral turpentine < toluene, while binary and ternary mixtures showed nonmonotonic impedance behavior, reflecting concentration-dependent intermolecular interactions. ANN models trained with the imaginary impedance component (*Z*″) demonstrated improved performance when restricted to the semicircle region of the Nyquist plots. This approach resulted in no misclassifications in the test set for adulterant type and enhanced regression performance for individual adulterants, even in mixed systems (test-set *R* ^2^ = 0.846 for *n*-hexane, 0.965 for toluene, and 0.742 for mineral turpentine). These results show that focusing on the semicircle domain concentrates the most informative impedance features and enables reliable identification and quantification of gasoline adulteration while also revealing the inherent complexity of multiadulterant fuel systems.

## Graphical abstract

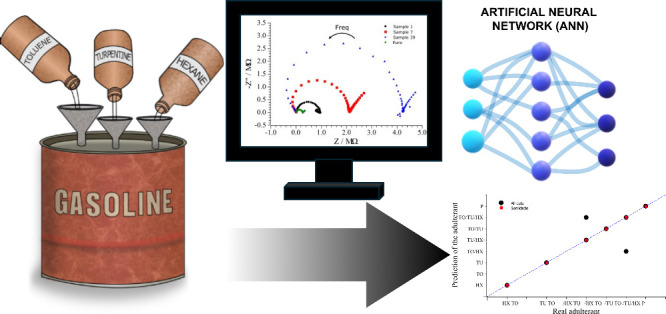

## 1 Introduction

Fuel quality control remains a global concern due to the wide range of fraudulent practices that compromise fuel integrity.^1–3^ Among the most common forms of adulteration is the illegal addition of organic solvents, such as toluene, *n*-hexane, and mineral turpentine, to gasoline, motivated by the pursuit of economic profit.^4^ Fuel adulteration has been reported in several countries and can significantly impact engine performance, consumer rights, air quality, and government tax revenues.^5,6^ In Brazil, for instance, the National Agency for Petroleum, Natural Gas and Biofuels (ANP) reported that in the last five years 1.6% of gasoline and 1.9% of hydrated ethanol samples were found to be noncompliant with regulatory standards, including adulteration cases.^7,8^ Although these percentages may appear small, the economic and environmental impact is substantial when considering the scale of the Brazilian market, more than 44 million cubic meters of gasoline were sold in 2024.^9^

The primary driver behind fuel adulteration is the disparity in taxation between different petroleum-derived products. Fraudsters exploit these differences by blending lower-taxed substances into higher-taxed fuels, often without immediate detection.^10^ In practice, the most common adulterants include lower-grade gasoline added to premium gasoline, diesel diluted with light heating oil, and gasoline mixed with cheaper petroleum-derived solvents such as kerosene and industrial solvents, including toluene, mineral turpentine, and *n*-hexane.^11^ To make matters worse, noncompliant fuels are typically sold at prices close to genuine products, making the fraud even more lucrative.

Common laboratory techniques for identifying adulteration include distillation (to assess boiling point profiles), colorimetric measurements, and advanced instrumental methods such as Raman spectroscopy, Fourier transform infrared spectroscopy (FTIR), ultraviolet–visible spectroscopy (UV–vis), gas chromatography, and high performance liquid chromatography (HPLC).^12–18^

Given the limitations of conventional analytical approaches, there is growing interest in developing more accessible, low-cost, and rapid screening techniques with comparable accuracy.^19^ Electrochemical sensors offer a promising alternative by enabling real-time monitoring.^20^ One particularly useful technique is electrochemical impedance spectroscopy (EIS), which is sensitive to changes in the electrical properties of the medium. EIS involves applying a small-amplitude AC signal across a range of frequencies using an electrochemical cell, where impedance values are calculated for each frequency and results are typically interpreted using Nyquist and Bode plots that reflect the interfacial and bulk properties of the system. This method can reveal subtle changes related to charge transfer, electrolyte resistance, and interfacial capacitance, making it well-suited for detecting low levels of contaminants.^21^

A few studies have shown that EIS parameters are strongly affected by physicochemical changes in biodiesel and gasoline matrices, enabling the discrimination of biodiesels produced from different feedstocks^22^ and supporting the detection of contaminants such as diesel and soot in lubricating oils.^23^ EIS has also been proposed as a fast, low-cost method for determining biodiesel content in diesel blends,^24^ as well as for quantifying water content in biodiesel.^25^ In addition, EIS-based electrical parameters have been correlated with the oxidative degradation of biodiesel^26^ and with aging processes in gasoline containing biocomponents.^27^

To enhance selectivity and interpretability, bioinspired sensor systems such as electronic tongues (ETs) have been developed for complex liquid analysis.^28^ An ET typically consists of low-selectivity sensors combined with data processing tools, based on chemometric analysis, such as principal component analysis (PCA) and artificial intelligence (AI) techniques such as artificial neural networks (ANNs).^29,30^ These systems can distinguish patterns in multicomponent mixtures, such as those found in fuel adulteration scenarios. Although the present work employs a single glassy carbon electrode as the working electrode, the approach adopted here follows the concept of a virtual electronic tongue. In this configuration, multiple orthogonal sensory channels are generated from a single physical sensor by applying distinct electrochemical perturbations or by exploring different regions of the signal domain. In the case of impedance spectroscopy, each frequency interval reflects a different physicochemical process at the electrode–solution interface, effectively producing a multisensor data set in the frequency domain.^28,31,32^

Only a few studies have applied electronic tongues to fuel analysis and adulteration monitoring. Bueno and Paixão developed an electrochemical tongue based on a copper interdigitated electrode and chemometric analysis for detecting water adulteration in ethanol fuel,^33^ while Souza et al. proposed a voltammetric system using carbon, gold, and platinum electrodes to discriminate gasoline, ethanol, biodiesel, and adulterated mixtures using chemometric analysis.^34^ Despite these advances, a review on portable forensic devices^35^ highlights that electrochemical multisensor systems are still rarely explored for hydrocarbon-based fuels. Meanwhile, reviews on biofuel analysis using other techniques^4,36,37^ reinforce the growing interest in pattern recognition and machine-learning approaches for this purpose.

Specifically regarding ANNs, they have been used mainly in combination with spectroscopic techniques in the fuel sector. They have been used to accurately predict physicochemical properties of biodiesel,^38^ estimate water content in biodiesel–diesel blends,^39^ and classify biodiesel feedstocks and blended fuels.^40^ ANN-based models have also shown superior performance compared to classical multivariate methods for detecting adulteration in diesel/biodiesel blends using vibrational spectroscopy^41^ and for predicting biodiesel purity.^42^

ANNs are particularly advantageous for interpreting EIS data because they can learn nonlinear and multivariate relationships directly from the full spectral response, without relying on an a priori equivalent-circuit model. This capability is especially important for complex chemical matrices, where forcing linear models often leads to imprecision. Consistent with this, previous studies have shown that ANNs extract information from nonlinear electrochemical signals more effectively than traditional linear chemometric tools, improving prediction and classification performance in multicomponent systems.^43,44^ Their architecture, comprising input, hidden, and output layers, enables the extraction of complex patterns that traditional statistical methods fail to capture.^43^

Few studies have combined electrochemical impedance spectroscopy (EIS) with artificial neural networks (ANNs) to enhance pattern recognition and quantitative modeling in complex matrices. Examples include the simultaneous quantification of alkali ions using EIS coupled to ANN models in fertilizer samples.^44^ Subsequent works have explored this integration for quality control and agri-food monitoring, such as ethanol quantification in pineapple waste^45^ and freeze-damage detection in tangerines.^46^ Moreover, ANN-EIS frameworks have been successfully applied in environmental sensing for pollutant classification.^47^

To the best of our knowledge, no previous study has integrated EIS and ANN in a format specifically designed for hydrocarbon-based fuels. Existing EIS–ANN applications are mostly limited to aqueous or agri-food systems and do not address the challenges of nonpolar, multicomponent gasoline matrices. Moreover, studies focused on detecting fuel adulteration typically rely on conventional chemometric tools, with no use of ANN-based EIS analysis. In this context, the present work combines electrochemical impedance spectroscopy (EIS) and artificial neural networks (ANNs) to advance the development of data-driven virtual electronic tongues for the discrimination of gasoline adulterated with organic solvents, while complementary Fourier-transform infrared spectroscopy (FTIR) measurements are used to provide supportive insight into the chemical interactions associated with the observed electrochemical responses.

## 2 Experimental Section

### 2.1 Materials and Reagents

All reagents used in this study were of analytical grade. For electrode characterization, potassium chloride (KCl, Merck, Germany) and potassium ferricyanide (K_3_[Fe­(CN)_6_], CARLO ERBA, Italy) were used. Solutions were prepared using deionized water with a resistivity of approximately 18.3 MΩ·cm, obtained from a Millipore Direct-Q 3 water purification system (Merck, Germany). For the preparation of adulterated fuel samples, type C gasoline was supplied by the Laboratory of Fuels and Petroleum Derivatives (LABCOM) at the School of Chemistry, Federal University of Rio de Janeiro (UFRJ). The adulterants used were toluene (Merck, Germany), *n*-hexane (Tedia, Brazil), and mineral turpentine (ITAQUA, Brazil).

### 2.2 Instrumentation

Cyclic voltammetry (CV) and electrochemical impedance spectroscopy (EIS) measurements were performed using a MultiAutolab M204 potentiostat/galvanostat (Metrohm Autolab, Netherlands), controlled by a laptop computer running Nova 2.1 software. A conventional three-electrode electrochemical cell was used, consisting of a glassy carbon working electrode, a platinum wire counter electrode, and a platinum wire pseudoreference electrode, with measurements performed under quiescent conditions. The glassy carbon surface was polished before each measurement using 0.05 μm alumina slurry and rinsed with deionized water. No electrochemical pretreatment was performed. Fourier-transform infrared spectroscopy (FTIR) analyses were performed using a Shimadzu IRAffinity-1 spectrophotometer equipped with a PIKE MIRacle single-reflection attenuated total reflectance (ATR) accessory with a ZnSe crystal, at a spectral resolution of 1 cm^–1^.

### 2.3 Fourier-Transform Infrared Spectroscopy (FTIR)

Prior to comparison, the FTIR spectra were normalized by setting the maximum absorbance intensity to one.

### 2.4 Cyclic Voltammetry

Cyclic voltammetry (Figure S1) was performed exclusively to verify the integrity and reproducibility of the electrochemical system prior to EIS measurements. CVs were recorded in a standard solution containing 0.01 mol·L^–1^ K_3_[Fe­(CN)_6_] and 0.1 mol·L^–1^ KCl, using a 2 mm diameter glassy carbon disk as the working electrode. Voltammograms were collected in triplicate over a potential window of −0.6 to +0.8 V at a scan rate of 25 mV·s^–1^. These measurements were used solely as an internal quality-control procedure to confirm electrode performance and absence of fouling prior to EIS and were not intended for analytical interpretation.

### 2.5 Electrochemical Impedance Spectroscopy (EIS)

All 59 fuel samples (nonadulterated and adulterated, as listed in Table S1) were analyzed using EIS. Measurements were carried out over a frequency range from 10^5^ Hz to 0.1 Hz, using a 10 frequency per decade distribution and a sinusoidal perturbation of 0.06 V_RMS_, relative to the open-circuit potential (OCP). Each sample was analyzed in triplicate, giving a total of 177 measurements. The data were presented as Nyquist plots, with *Z*′ and *Z*″ corresponding to the real and imaginary components of the impedance (in ohms), respectively.

### 2.6 Sample Preparation

The gasoline used in this study was obtained from commercial fuel stations and therefore corresponded to gasoline blended with anhydrous ethanol, as required by Brazilian regulations. Samples referred to as nonadulterated gasoline correspond to this commercial fuel and contained 27% (v/v) anhydrous ethanol. Consequently, the adulterated samples also contained ethanol.

A total of 54 adulterated samples (samples 1 to 54) were prepared by volumetric addition of mineral turpentine, *n*-hexane, and toluene at concentrations of 0.0%, 3.0%, 7.0%, and 10.0%. All possible combinations of these levels were generated while constraining the total adulterant content to 10–30% (v/v), following a restricted full-factorial design rather than a one-factor-at-a-time approach. Each sample had a final volume of 10.00 mL. The detailed composition of all samples is provided in Table S1. For clarity throughout the text, the following coding format will be used to identify each sample composition:

(%v/v)TO_(%v/v)TU_(%v/v)HX

where TO = toluene, TU = mineral turpentine, and HX = *n*-hexane.

For example, Sample 28 contains 7% toluene, 3% mineral turpentine, and 3% *n*-hexane and is therefore coded as 7TO_3TU_3HX.

Additionally, five nonadulterated gasoline samples were analyzed.

### 2.7 Data Analysis and Modeling Using Artificial Neural Networks (ANNs)

For ANN modeling, only the imaginary component of the impedance (*Z*″) obtained at all measured frequencies was used. These *Z*″ values were directly exported from the potentiostat software and arranged into feature vectors representing each sample, without any fitting procedures or extraction of electrochemical parameters.

Data analysis and modeling were performed using Orange Data Mining software, version 3.38.1. The data set was split into training (80%) and testing (20%) subsets. In addition, a stratified 5-fold cross-validation was applied to ensure the robustness of the model, preserving the class distribution and allowing each sample to be used for both training and validation across folds. To further confirm model stability, repeated random sampling was also performed (10 repetitions with 66% of the data used for training in each iteration). Before model training, a preliminary optimization was performed to define the most suitable ANN architecture. Different numbers of neurons (100–1200), activation functions (Identity, Logistic, tanh, ReLU), and solvers (L-BFGS, SGD, Adam) were systematically evaluated. Model performance was assessed using the area under the ROC curve (AUC), classification accuracy (CA), F1-score, precision, and recall for classification tasks, and MSE (mean squared error), RMSE (root mean squared error), MAE (mean absolute error), and *R* ^2^ for regression outputs (the evaluation parameters used to assess model performance are better described in the Supporting Information). Modeling was conducted using the Neural Network widget, optimized configuration with 900 neurons in the hidden layers, the tanh activation function, and the L-BFGS solver.

Regarding computational requirements, Orange Data Mining was used as a development environment. Once trained, the ANN performs inference through a simple forward propagation step, requiring low computational cost. Inference was carried out on a standard CPU-based system without GPU acceleration, with prediction times per sample on the order of seconds, indicating suitability for real-time and portable applications.

## 3 Results and Discussion

### 3.1 Impact of Adulterants on Fuel Properties and Engine Performance

*n*-Hexane is a light aliphatic hydrocarbon with a low octane number. Its addition to gasoline tends to reduce the overall octane rating of the fuel blend, which can lead to engine knocking and diminished performance. Due to its high volatility, *n*-hexane may evaporate rapidly or infiltrate the engine’s lubricant system, diluting the oil and impairing its protective properties. This can accelerate engine wear and damage. Furthermore, the incomplete combustion of *n*-hexane-adulterated gasoline increases the emission of unburned hydrocarbons, contributing to environmental pollution. Industrial-grade *n*-hexane may also contain sulfur and acidic compounds, increasing fuel corrosiveness.

Toluene, while commonly used as an octane booster and naturally present in regulated amounts in commercial gasoline, can be problematic when added in excess. Although it increases the octane rating, high concentrations of toluene can disrupt the ideal air–fuel ratio, hinder vaporization, and lead to incomplete combustion. These effects may result in reduced engine efficiency, higher fuel consumption, and deposit formation on fuel injectors and valves. Inadequate combustion of toluene also leads to increased emissions of toxic pollutants, including unburned hydrocarbons, carbon monoxide, and particulate matter, thereby worsening air quality and posing risks to human health.

Mineral turpentine, a complex mixture of aliphatic and aromatic hydrocarbons, is not formulated for combustion engine use. Its addition to gasoline significantly alters key fuel properties such as octane number, volatility, and chemical stability. As a low-octane solvent, mineral turpentine can decrease combustion efficiency, leading to engine knocking, misfires, power loss, and increased wear on components such as spark plugs, valves, and pistons. Its high tendency to form gums and residues over time can cause clogging and operational issues. Moreover, mineral turpentine can infiltrate the lubricant system and accelerate oil degradation. Being an industrial solvent, it frequently contains corrosive substances that may dry out or damage rubber seals and hoses in the fuel delivery system.

In practice, gasoline adulteration typically occurs within the 10–30% v/v range, as lower additions provide little economic benefit to the adulterer, whereas higher levels tend to cause noticeable engine performance issues that facilitate detection. More importantly, adulterations within this interval are particularly difficult to identify using conventional analytical methods, as noted by Mabood et al.,^11^ who reported that “detection of gasoline adulteration, especially when it is with lower percentage (10–30% by volume), cannot be easily done”. For this reason, the concentration levels adopted in this study (0%, 3%, 7%, and 10% v/v), and their structured combinations, were selected to reproduce realistic multiadulterant scenarios that fall within this critical detection window. A subset of samples was selected for discussion in the following sections to avoid redundancy while covering all relevant scenarios. Nonadulterated gasoline and the three single-adulterant samples at 10% v/v were included to show the individual effect of each solvent. Representative binary and ternary mixtures were chosen because they capture the main interaction patterns observed in the data set. Together, these selected samples provide a concise yet representative overview of the system’s behavior.

### 3.2 Electrochemical Impedance Spectroscopy (EIS): Interfacial and Molecular Insights

#### 3.2.1 Nonadulterated Gasoline and Single-Adulterant Samples

The impedance response of nonadulterated and adulterated gasoline was examined with complementary spectroscopic evidence integrated to support the interpretation of interfacial phenomena. The addition of organic solvents and oxygenated compounds is known to modify bulk physicochemical properties of gasoline, such as polarity, dielectric behavior, and molecular organization, which can indirectly affect the electrode–solution interface and the measured impedance response. Initially, a conventional Ag/AgCl reference electrode was adopted as reference electrode. However, due to the organic nature of the fuel samples, the aqueous KCl-based Ag/AgCl reference electrode proved inadequate, resulting in unstable potentials caused by incompatibility between the aqueous filling solution and the nonpolar fuel matrix. To address this issue and improve measurement stability, the Ag/AgCl electrode was replaced with a platinum wire pseudoreference electrode, minimizing interfacial mismatches.

All EIS experiments were performed at the open-circuit potential (OCP). This choice was made to avoid polarizing the working electrode in a way that depends on sample composition. For pure gasoline, the OCP measured in triplicate was 0.0947 ± 0.012 V, whereas sample 10 (0TO_10TU_10HX) exhibited a significantly different average OCP of −0.097 ± 0.008 V. If a fixed reference potential (e.g., 0 V vs ref.) had been applied to all samples, pure gasoline would have been subjected to anodic polarization while sample 10 would have experienced cathodic polarization, thereby introducing an additional and undesired source of variability in the impedance response.

The presence of a semicircle in a Nyquist plot is typically associated with an electrochemical interface consisting of an electrolyte, characterized by a solution resistance (*R*_S_), obtained from the high-frequency intercept with the real axis; the double-layer capacitance (*C*_DL_), which defines the frequency of the maximum imaginary contribution in the total impedance; and the charge-transfer process represented by the charge-transfer resistance (*R*_CT_), calculated from the semicircle diameter. Typically, after the semicircle an approximately linear behavior is observed, i.e., an increasing impedance trend typically associated with diffusion-controlled processes represented by a Warburg-type impedance element. For a more detailed and visual explanation on the Nyquist plot, please refer to ref 21.

Figure 1 presents the Nyquist plots obtained from three independent measurements of nonadulterated gasoline using freshly cleaned glassy carbon electrodes The three independent measurements were performed to evaluate system suitability and to verify the stability of the baseline response prior to analyzing adulterated samples. The three curves exhibit consistent impedance profiles, showing a level of reproducibility that is appropriate for measurements carried out in a highly resistive, nonpolar organic medium. To quantitatively assess the reproducibility, key electrochemical parameters were extracted from each measurement: *R*_S_, *C*_DL_, and *R*_P_. The relative standard deviations (%RSD) for these parameters were: −14.2, 2.0, and 2.6%, respectively. This stability is essential for ensuring that subsequent differences observed between nonadulterated and adulterated gasoline arise from genuine compositional variations rather than instrumental drift or electrode conditioning effects.

**Figure 1:**
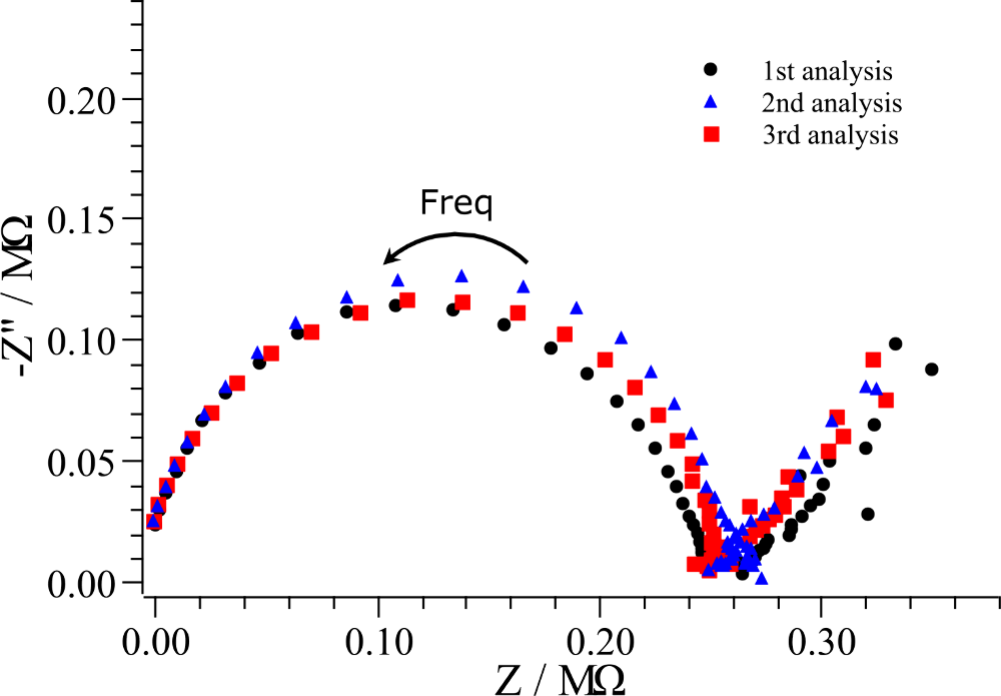
Electrochemical impedance spectroscopy (EIS) of three independent measurements of nonadulterated gasoline recorded with a glassy carbon working electrode and two platinum wires serving as reference and counter electrodes. Measurements were carried out over a frequency range from 10^5^ Hz to 0.1 Hz, using a 10 frequency per decade distribution and a sinusoidal perturbation of 0.06 VRMS, relative to the open-circuit potential (OCP).

Gasoline is composed primarily of weakly polarizable molecules, which results in a low dielectric constant and a limited ability to promote salt dissociation.^48,49^ Consequently, the low concentration of free charge carriers leads to high impedance values, considerably greater than those of water-based samples and consistent with the inherently low conductivity of nonadulterated gasoline. In Brazilian commercial gasoline, which contains up to 27% (v/v) ethanol, this behavior can be modified. The influence of ethanol on impedance measurements arises from both its conductive and electrochemical properties. Ethanol has a higher dielectric constant than gasoline, which increases the overall conductivity of the medium, and it is also a redox-active specie capable of undergoing oxidation at the electrode surface. Consequently, impedance values at higher frequencies tend to be lower than those of ethanol-free gasoline, while the semicircular shape of the Nyquist plot is influenced by the charge-transfer resistance associated with ethanol oxidation.^50^

Figure 2 displays the EIS results for the nonadulterated gasoline and samples 1, 7, and 39 (0TO_0TU_10HX, 0TO_10TU_0HX, and 10TO_0TU_0HX, respectively).

**Figure 2:**
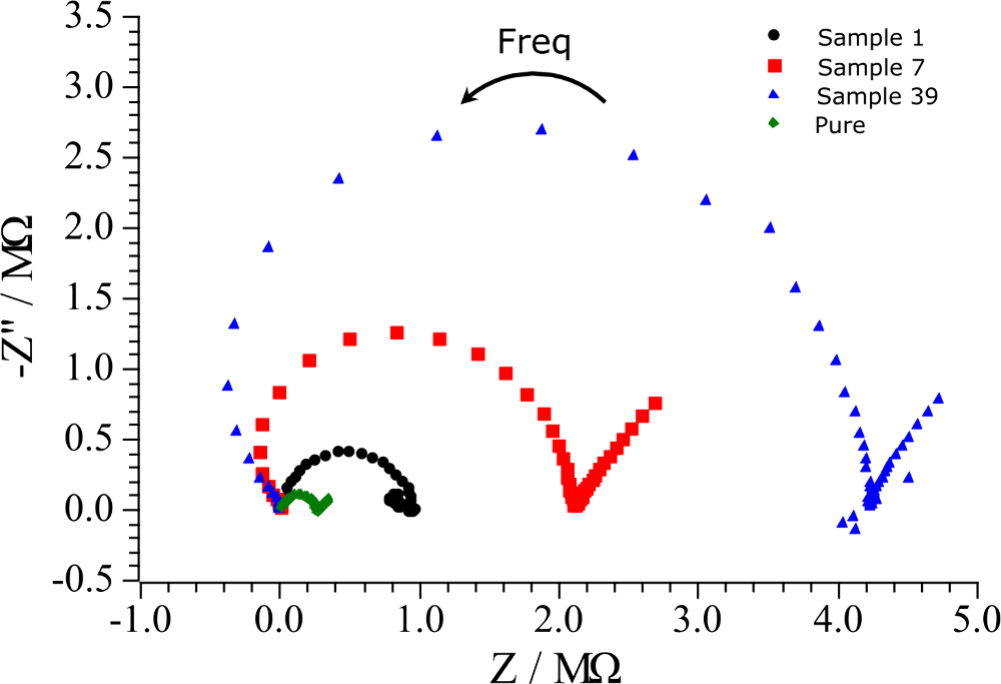
EIS analysis of gasoline samples not adulterated (pure - diamond green) and adulterated: 0TO_0TU_10HX (sample 1 – black circle), 0TO_10TU_0HX (sample 7 – red square), and 10TO_0TU_0HX (sample 39 – blue triangle). Glassy carbon was used as the working electrode, and two platinum wires were used as reference and counter electrodes. Measurements were carried out over a frequency range from 10^5^ Hz to 0.1 Hz, using a 10 frequency per decade distribution and a sinusoidal perturbation of 0.06 VRMS, relative to the open-circuit potential (OCP).

Comparing the Nyquist plot of the gasoline not adulterated and adulterated (Figure 2), the semicircle for nonadulterated gasoline (pure gasoline) is approximately 1 order of magnitude smaller than those of the adulterated samples. Additionally negative impedance is observed for the adulterated samples. Although uncommon, negative real impedance values have been reported in the literature.^51–53^ Negative real impedance can occur under specific conditions in which the current decreases as the potential increases, an indication of electrochemical instability that leads to negative faradaic impedance. According to Koper M.T.M and Sluyters J.H.,^54^ negative faradaic impedance may arise in four situations: (i) Available electrode surface decreases with increasing polarization; (ii) Potential-dependent adsorption of an inhibitor; (iii) Potential-dependent desorption of a catalyst and (iv) Electrostatic effect at low ionic strength. The last scenario consists of the measurements in this article, which were conducted in a medium with very low conductivity. A deeper investigation of this phenomenon lies beyond the scope of the present work; for this reason, our discussion of the EIS results focuses on a qualitative interpretation of the data.

For the adulterated samples the Nyquist plots revealed an increase in semicircle diameter in the order *n*-hexane < mineral turpentine < toluene. On the other hand, all samples exhibited similar high-frequency intercepts, indicating that changes in *R*_CT_ were more significant than those in *R*_S_. This behavior can be explained by the adsorption of the adulterant molecules on the glassy carbon electrode, which hinders electron transfer and increases *R*_CT_. Glassy carbon combines amorphous domains with graphitic regions containing sp^2^-hybridized carbons. Among the adulterants, toluene, an aromatic solvent, can adsorb strongly through π–π dispersion interactions with the delocalized electrons of sp^2^ carbon atoms Mineral turpentine, in contrast, is a complex mixture of organic compounds that allows some degree of π–π interaction, though weaker and less consistent than in the case of toluene, but stronger than the pure aliphatic *n*-hexane, which explains the smaller diameter observed in Nyquist plots for *n*-hexane.^55,56^

#### 3.2.2 Gasoline Adulterated with Ternary Adulterant Mixtures

Although the effect of each individual adulterant can be correlated with changes in the impedance spectra, the situation becomes more complex when multiple adulterants are present in the same sample. To illustrate this effect, Figure S2 shows Nyquist plots obtained when all three adulterants were added to the same sample in equal proportions at increasing concentrations. As the gasoline content decreases from 91% (sample 13–3TO_3TU_3HX) to 79% (sample 33–7TO_7TU_7HX), the higher concentration of adsorbing molecules leads to a larger semicircle diameter. However, when the gasoline content decreases further to 70% (sample 54–10TO_10TU_10HX), the semicircle diameter decreases, evidencing an oscillatory electrochemical response rather than a monotonic concentration effect.

This oscillatory impedance behavior is supported by FTIR evidence (Figure S4), which reveals concentration-dependent oscillations in key spectral markers associated with molecular availability and organization. In the O–H overtone region, the band center shifts from 3339 cm^–1^ in the baseline gasoline–ethanol blend to 3346 cm^–1^ in Sample 33, accompanied by a narrowing of the band (Full width at half-maximum (fwhm) = 316 → 275 cm^–1^), indicating an increase in disrupted but relatively homogeneous hydrogen-bond environments. At higher adulterant concentration (Sample 54), although the band remains blue-shifted (3347 cm^–1^), the fwhm increases again (291 cm^–1^), evidencing a return to a more heterogeneous hydrogen-bonding regime. This shift–narrowing–broadening sequence explicitly demonstrates an oscillatory reorganization of the molecular environment.

A second oscillatory signature is observed in the aromatic fingerprint region. In the 600–800 cm^–1^ window, the characteristic out-of-plane C–H bending band shifts from approximately 678 cm^–1^ toward 676 cm^–1^ at intermediate adulteration (Sample 33) and returns toward 678 cm^–1^ at higher adulterant levels (Sample 54). This 678 → 676 → 678 cm^–1^ sequence indicates oscillations in the local environment and association state of aromatic species, reflecting changes in the number of molecules effectively available at the electrode interface.

Taken together, the oscillatory trends observed independently in the O–H overtone region and in the aromatic fingerprint bands provide spectroscopic evidence that the number and organization of surface-accessible species vary nonmonotonically with adulterant concentration. The impedance response, therefore, captures these oscillations in molecular availability more clearly, while FTIR reveals subtle, concentration-dependent spectroscopic signatures of the same reorganization, increasing when dispersed species dominate and decreasing when enhanced intermolecular interactions and microdomain formation limit access to the electrode surface.

#### 3.2.3 Gasoline Adulterated with Binary Adulterant Mixtures

To evaluate the effect of each adulterant without the influence of gasoline dilution, Figures S5, S6, and S8 present binary adulterant mixtures in which the total adulterant content remains approximately constant while the relative proportions vary.

Figure S5 shows mixtures of toluene and mineral turpentine with *n*-hexane fixed at 0%. The largest semicircle occurred in Sample 43 (10TO_3TU_0HX). As mineral turpentine increased and toluene decreased, the semicircle diameter progressively diminished, as observed in Sample 31 (7TO_7TU_0HX) and Sample 20 (3TO_10TU_0HX). In this system, reducing mineral turpentine and increasing toluene results in larger semicircles, in accordance with the observed behavior for the single adulterated samples in Figure 2.

Figure S6 highlights mixtures of *n*-hexane and toluene with mineral turpentine fixed at 0%. Between Sample 12 (3TO_0TU_10HX) and Sample 25 (7TO_0TU_7HX), raising toluene from 3% to 7% while lowering *n*-hexane from 10% to 7% markedly increased the semicircle diameter, indicating that the impedance response becomes increasingly governed by toluene. This agrees with the single-adulterant results in Figure 2, where toluene exhibited the largest semicircle. However, this tendency reversed at higher toluene and lower *n*-hexane contents: in Sample 40 (10TO_0TU_3HX), the semicircle became slightly smaller than at the intermediate mixture, suggesting reduced availability of toluene for surface adsorption due to enhanced intermolecular interactions.

FTIR data support this interpretation by revealing composition-dependent changes in toluene organization within the mixture in Figure S7. In Sample 40 (10TO_0TU_3HX), the band near 678 cm^–1^, associated with aromatic ring vibrations, becomes more pronounced and shifts toward values characteristic of neat toluene, indicating increased toluene–toluene association. Concomitantly, subtle shifts in the aromatic C–H stretching region (3027–3030 cm^–1^) and changes in band shape due to overlap with aliphatic C–H modes reflect variations in the local molecular environment as the *n*-hexane fraction decreases. Together, these spectral features are consistent with enhanced intermolecular interactions among toluene molecules at higher toluene contents, which reduce their effective availability for adsorption at the electrode surface and account for the slight decrease in the Nyquist semicircle observed in Sample 40.

Figure S8 exhibits mixtures of *n*-hexane and mineral turpentine while toluene was kept at 0%. The largest semicircle refers to Sample 3 (0TO_3TU_10HX), which contains the higher amount of *n*-hexane and the lower amount of mineral turpentine. As the proportion of mineral turpentine increases and *n*-hexane decreases, the semicircle shrinks, indicating lower *R*_CT_. In this binary the dominant factor does not appear to be adsorption strength, since the opposite behavior would be expected (increase of *R*_CT_ with lower amount of *n*-hexane and higher amount of mineral turpentine).

Spectroscopic characterization of mineral turpentine reveals the presence of O–H-related vibrational features (Figure S3), indicating oxygenated functionalities capable of interacting with the ethanol-containing fuel matrix. Such interactions can indicate a preferential association of mineral turpentine within the bulk phase, which may reduce the fraction of molecules effectively available for interfacial adsorption as its concentration increases. In more *n*-hexane-rich mixtures, this association is likely attenuated, leaving a larger fraction of mineral turpentine molecules accessible for interfacial interaction, in agreement with the larger Nyquist semicircle observed at lower turpentine contents.

These findings demonstrate that the molecular nature of the adulterants and their concentration strongly affect the impedance signature of the fuel. They confirm the value of EIS as a diagnostic tool for detecting and differentiating types of adulteration, while also emphasizing its limitations in predicting composition when multiple adulterants coexist. To address this challenge, in the next section we introduce an analytical approach based on artificial neural networks (ANN) to identify the composition of adulterated samples.

### 3.3 Artificial Neural Networks (ANNs)

Following EIS measurements, all data were exported to an Excel spreadsheet and analyzed using Orange software for artificial neural network (ANN) modeling. To build predictive models, the data set was split into two subsets: 80% for training and 20% for testing. During training, the algorithm learned the relationships and patterns among the variables, while the remaining 20% of the data was reserved for evaluating the model’s generalization ability. Two different models were developed: (i) a classification model to determine whether the gasoline was adulterated and to identify the specific adulterant, and (ii) a regression model to quantify the adulterant concentration. Modeling was performed using the Neural Network widget, configured with 900 neurons in the hidden layers, the tanh activation function, and the L-BFGS solver. The tanh (hyperbolic tangent) activation function maps input values to an output range between −1 and 1, introducing nonlinearity into the model while maintaining zero-centered outputs, which can improve convergence during training. The L-BFGS (limited-memory Broyden–Fletcher–Goldfarb–Shanno) solver is a quasi-Newton optimization algorithm that uses an approximation to the Hessian matrix to find the optimal weights efficiently. It is particularly effective for smaller to medium-sized data sets and can converge faster and more reliably than stochastic gradient-based methods.^29,43^

The choice of the number of neurons, activation function, and solver was based on a preliminary evaluation of multiple combinations of activation functions (Identity, Logistic, tanh, ReLU) and solvers (L-BFGS, SGD, Adam), with this configuration providing the best results. For the classification models, performance was evaluated using the area under the ROC curve (AUC), classification accuracy (CA), F1-score, precision, and recall, where values closer to 1 indicate better performance. For the regression models, the evaluation considered the mean squared error (MSE), root mean squared error (RMSE), mean absolute error (MAE), and the coefficient of determination (*R* ^2^), where lower error values and *R* ^2^ values closer to 1 indicate better performance.^29,43^ The calculation of each metric is detailed in the Supporting Information.

Initially, all ANN models were constructed using the complete *Z*″ data from the impedance experiments. However, the results revealed greater dispersion of impedance values toward the end of the semicircle in all Nyquist plots (main text and Supporting Information). To enhance model precision, an alternative approach was adopted in which only the data corresponding to the semicircle region was used as input for the ANN models.

#### 3.3.1 Classification Model

Figure 3 presents the classification performance metrics for ANN models trained using two different input data sets: the complete *Z*″ impedance data and only the semicircle portion of the Nyquist plots. Results are shown for both the model fitting (Figure 3A) and the corresponding predictions on the test set (Figure 3B). It is important to highlight that the purpose of this model is strictly to identify the type of adulterant present in the sample, regardless of its concentration. Quantification is addressed separately by the regression models discussed in Section 3.3.2.

**Figure 3:**
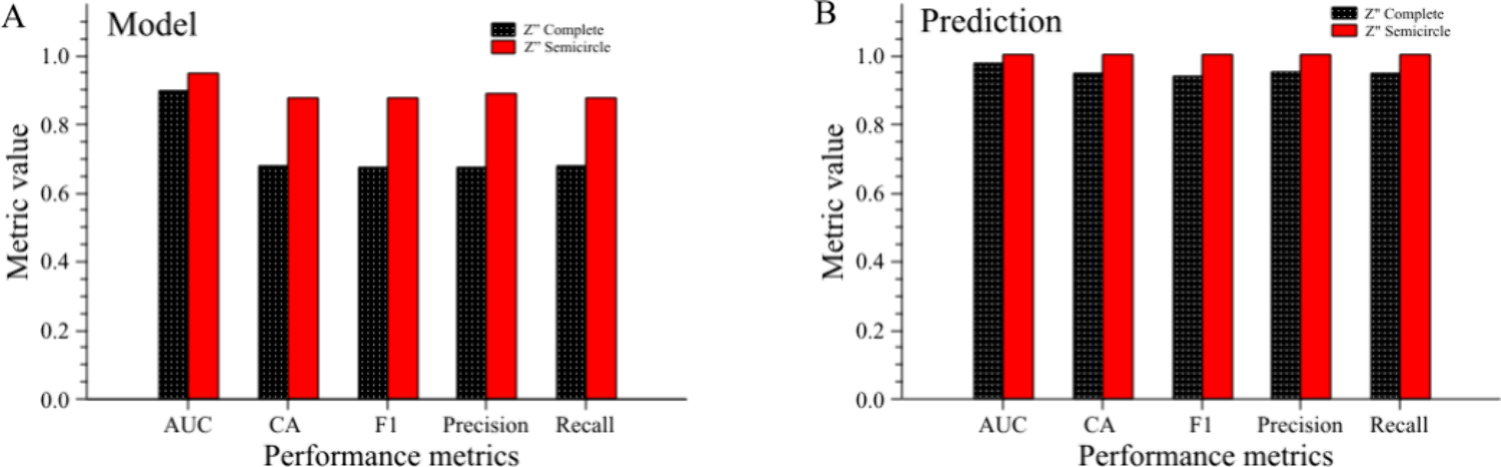
Performance metrics for the classification of ANN models using either the complete *Z*″ data set (black bars with white dotted pattern) or only the semicircle portion of the Nyquist plots (solid red bars). (A) “Model” refers to the training phase, and (B) “Prediction” refers to the test set results. AUC: Area Under the ROC Curve, CA: Classification Accuracy, F1: Harmonic mean of Precision and Recall, RECALL: Proportion of actual positive instances correctly identified by the model. For the metric formulas, please refer to the Supporting Information.

Restricting the input to the semicircle portion of the *Z*″ data resulted in a clear improvement across all metrics. Compared to the complete data set, the semicircle-based models achieved higher AUC, CA, F1-score, Precision, and Recall values in both training and prediction. In particular, the prediction metrics for the semicircle data reached perfect scores (1.000) for all parameters, indicating that removing the high-dispersion region at the end of the Nyquist plots reduced noise and enhanced the model’s discriminative capacity. This improvement can be attributed to the removal of the low-frequency portion of the impedance spectra, which is typically dominated by diffusion-related processes. By restricting the analysis to the semicircle region, which reflects molecular adsorption phenomena at the electrode surface, the ANN model focused on the most stable and reproducible features of the system.

Although the data set contains 59 samples, the dimensionality of the predictor space is low: the complete impedance data set comprises 61 *Z*″ features per sample, and the semicircle-only data set contains 30 features. These dimensionality levels are far from the high-dimensional regime in which the curse of dimensionality becomes relevant, i.e., when data requirements grow exponentially with the number of features. In the present system, the impedance patterns are simple, structured, and highly consistent within each class, allowing the ANN to learn the discriminative features effectively even with a modest data set. This assessment is supported by the independent test set, which reproduced the same high performance observed during training. The agreement between training and test metrics confirms that the models generalize well and that the perfect scores obtained for the semicircle data reflect intrinsic class separability rather than overfitting.

Figure 4 compares the real and predicted adulterant types for each individual test sample. The dashed diagonal line represents the ideal identity condition (predicted = real) and is included solely as a visual reference for perfect classification; it does not correspond to a fitted regression line. All data points shown correspond to independent test samples. When trained with the complete *Z*″ data set (black circles), the models exhibited some misclassifications, particularly for mixed-adulterant samples such as TU/HX and TO/TU/HX; consequently, samples with the same adulterant combination may appear both on and off the diagonal, reflecting correct and incorrect classifications of distinct samples rather than duplicated data points. In contrast, models using only the semicircle portion (red squares) aligned perfectly with the diagonal reference line, consistent with the perfect prediction metrics in Figure 3. These findings confirm that excluding the high-dispersion region substantially improves the ANN’s ability to correctly identify the adulterant type.

**Figure 4:**
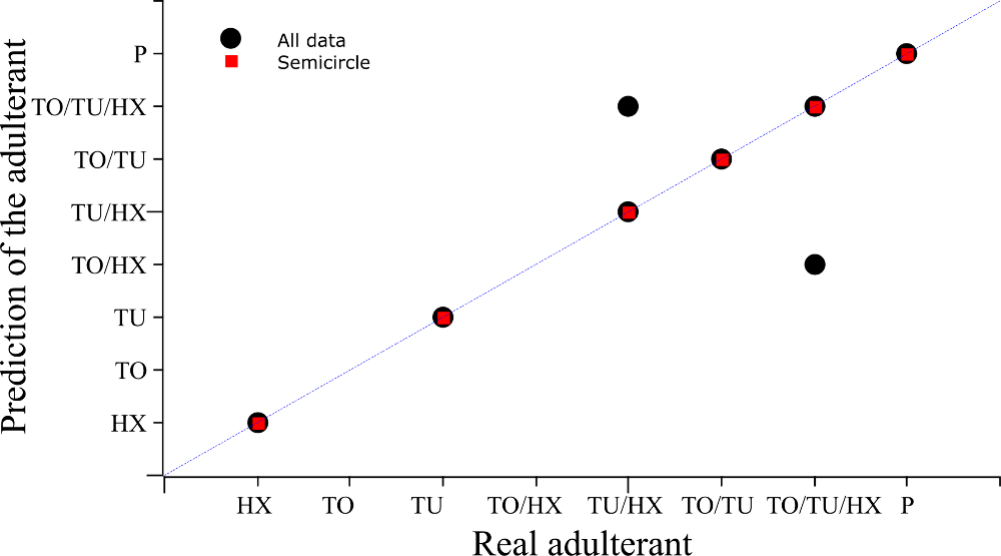
Comparison between the real and predicted types of adulterants for ANN models trained with the complete *Z*″ data set (black circles) and only the semicircle portion (red squares). HX = *n*-hexane; TO = toluene; TU = mineral turpentine; *P* = nonadulterated gasoline. The dashed diagonal line represents the ideal identity condition (predicted = actual) and is included solely as a visual reference for perfect classification, not as a fitted regression.

#### 3.3.2 Regression Models

Figure 5 summarizes the regression performance metrics obtained for each adulterant (*n*-hexane, toluene, and mineral turpentine) using two different input data sets: the complete *Z*″ impedance data and only the semicircle portion of the Nyquist plots. Results are presented separately for the training phase (Figure 5A - “Model”) and the prediction phase (Figure 5B - “Prediction”), allowing direct comparison of model fitting and predictive capability. In contrast to the classification model, the regression models are designed to estimate the concentration of each adulterant across all sample types, including single-adulterant, binary, and ternary mixtures, providing quantitative information that complements the identification step.

**Figure 5:**
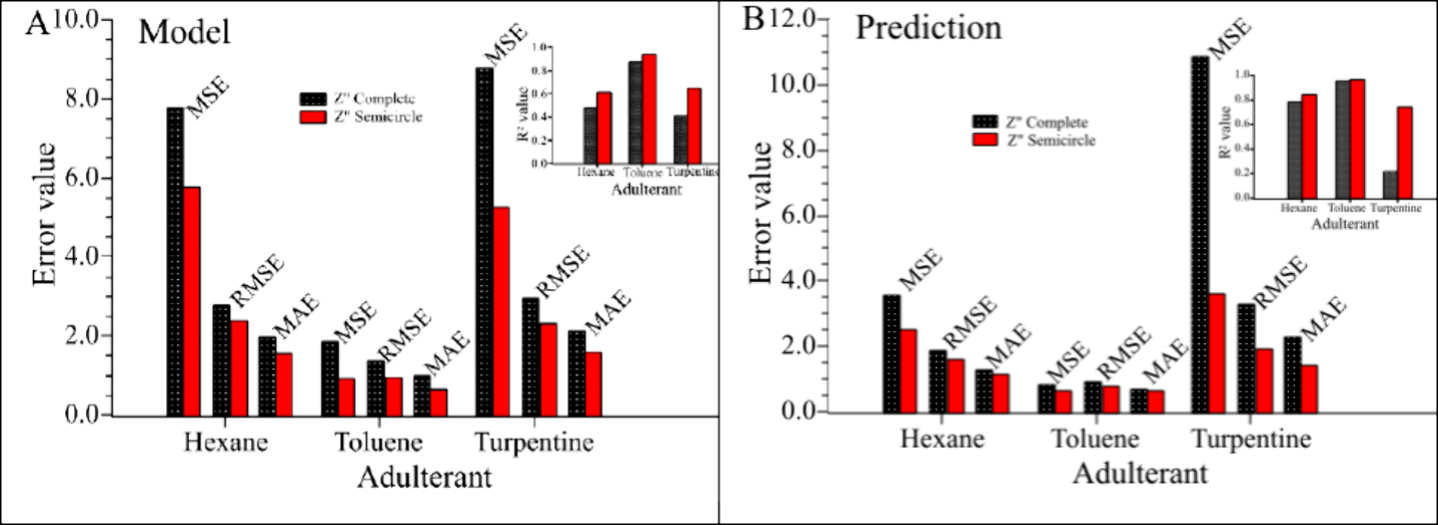
Regression performance metrics: MSE, RMSE, MAE, and *R* ^2^ (inset) for ANN models trained using the complete *Z*″ data set (black bars with white dotted pattern) and only the semicircle portion of the Nyquist plots (solid red bars). Results are shown for each adulterant (n-hexane, toluene, and mineral turpentine) in (A) the training phase (“Model”) and (B) in the prediction phase (“Prediction”). Lower error values (MSE, RMSE, MAE) and higher *R* ^2^ indicate better performance. MSE: mean squared error, RMSE: root mean squared error, MAE: mean absolute error, *R* ^2^: coefficient of determination. For the metric formulas, please refer to the Supporting Information.

In the training phase (Figure 5A), using only the semicircle portion (solid red bars) consistently improved performance across all adulterants, with lower error values (MSE, RMSE, MAE) and higher *R* ^2^ (inset) compared to the complete data set (black bars with white dotted pattern). Toluene achieved the best fit, with the lowest errors and the highest *R* ^2^ values (0.873 for complete data and 0.939 for semicircle data). *n*-Hexane and mineral turpentine also benefited from the semicircle approach, though with lower overall *R* ^2^ values, indicating a comparatively less accurate fit.

From a practical standpoint, the applicability of the proposed method is defined within the experimentally investigated concentration range. Robust classification and reliable quantitative performance were achieved for adulterant levels between 3 and 10% (v/v), which corresponds to the range most relevant for real-world gasoline adulteration.^11^

In the prediction phase (Figure 5B), the improvement with the semicircle data set was even more pronounced for all adulterants (the dashed diagonal line represents the ideal identity condition (predicted = actual) and is shown only as a visual reference). Toluene again presented the best performance, achieving the lowest prediction errors and *R* ^2^ values above 0.95. For *n*-hexane and mineral turpentine, the semicircle data reduced prediction errors substantially, with mineral turpentine showing the largest relative gain in *R* ^2^ (from 0.218 to 0.742).

These results reinforce that removing the high-dispersion end of the Nyquist curve increases the robustness of the models and their predictive capability, particularly for more challenging adulterants such as mineral turpentine. As discussed earlier, the impedance behavior of adulterated samples is mainly governed by adsorption phenomena that modulate the charge-transfer resistance (*R*_CT_). When the regression model is restricted to the semicircle region, dominated by charge-transfer phenomena, it captures these adsorption-related effects more accurately, reducing variability from less relevant regions (low-frequency region) of the spectrum and improving predictive performance.

For *n*-hexane, the regression models trained with only the semicircle data (Figure 6A – red square) produced predictions that were closer to the ideal 1:1 line, with reduced dispersion across all concentration levels. This improvement is particularly evident at intermediate and higher concentrations, where the complete data set tended to underestimate the real concentration and showed larger variability. The smaller error bars for the semicircle data confirm a more consistent model response, aligning with the lower prediction errors and higher *R* ^2^ reported in Figure 5.

**Figure 6:**
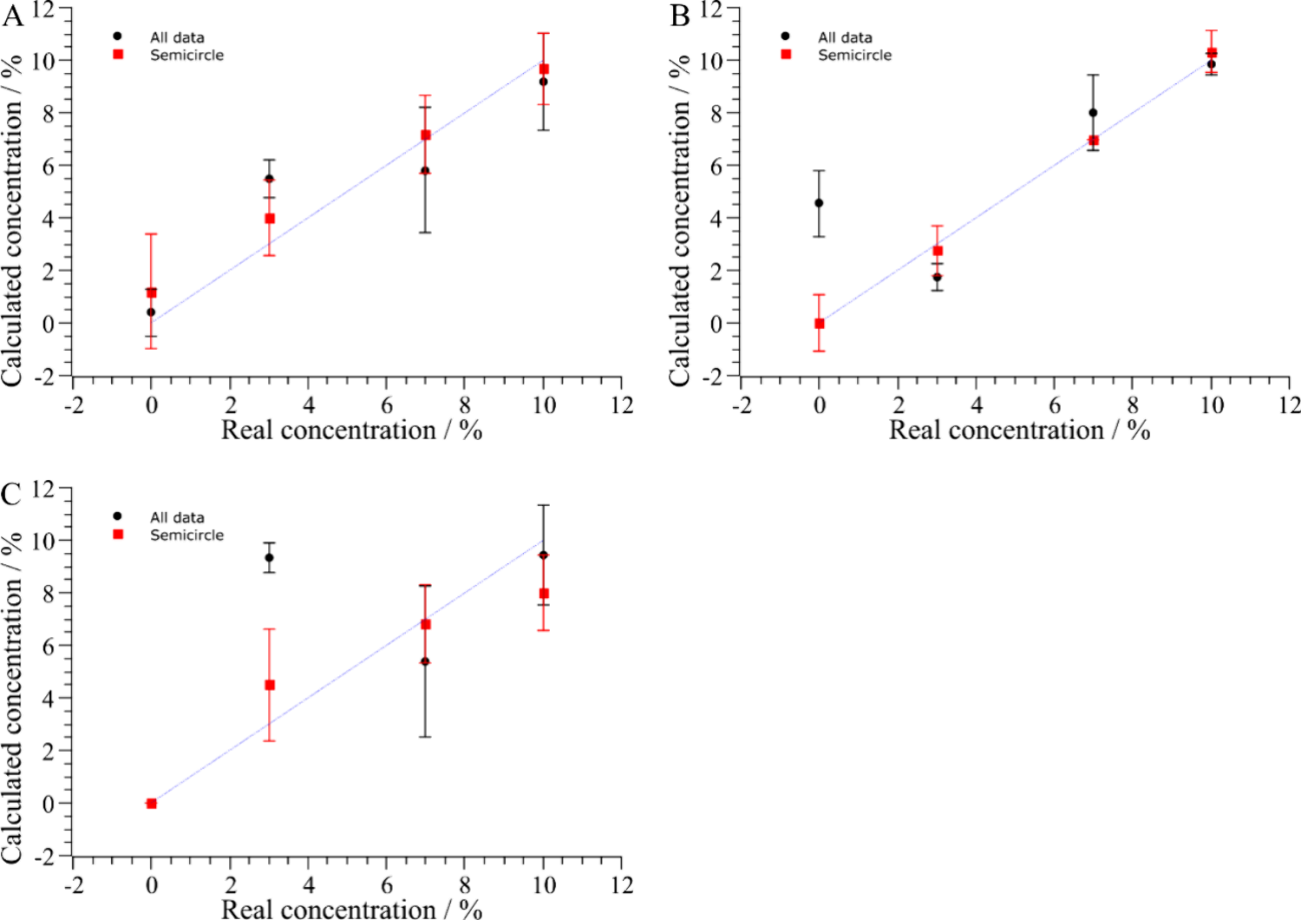
Predicted vs real concentrations of (A) *n*-hexane, (B) toluene, and (C) mineral turpentine for ANN regression models trained with the complete *Z*″ data set (black circles) and only the semicircle portion (red squares). Error bars represent standard deviations for triplicate measurements. The dashed diagonal line (1:1) represents the ideal identity condition (predicted = actual) and is included solely as a visual reference for perfect classification, not as a fitted regression.

For toluene (Figure 6B), both the complete *Z*″ and semicircle-based models produced predictions closely aligned with the 1:1 reference line, reflecting the high *R* ^2^ values obtained in the regression metrics. Nevertheless, the semicircle model slightly reduced the deviation at lower and intermediate concentrations and minimized variability, as shown by the smaller error bars. This confirms the superior predictive accuracy and consistency of the semicircle approach, particularly relevant for precise quantification at lower concentration ranges.

For mineral turpentine (Figure 6C), the model trained with the complete *Z*″ data set exhibited substantial deviations from the 1:1 reference line, particularly at low and intermediate concentrations, where overestimation and increased variability were observed. In contrast, the semicircle-based model markedly improved alignment with the ideal prediction line and reduced the dispersion, especially for the intermediate concentration range. This improvement is consistent with the significant increase in *R* ^2^ (from 0.218 to 0.742) reported in Figure 5, highlighting the benefit of excluding the high-dispersion portion of the Nyquist data for this more challenging adulterant.

Across all adulterants, using only the semicircle portion of the *Z*″ data consistently improved predictive performance, as reflected by closer alignment with the 1:1 line and reduced variability in the predicted concentrations. Toluene showed the best overall performance with minimal deviations for both data sets, while *n*-hexane and especially mineral turpentine benefited the most from the semicircle approach, with marked reductions in error and variability.

From a practical perspective, the proposed methodology also opens opportunities for future simplification and modernization. The reliance on impedance features that do not require equivalent circuit fitting, combined with data-driven modeling, makes the approach well suited for translation into sensor-based platforms. Future developments may involve the integration of miniaturized electrodes, simplified impedance acquisition hardware, and embedded machine learning models, enabling rapid and user-friendly quantification of gasoline adulterants in field or regulatory screening applications.

## 4 Conclusions

This work demonstrates the potential of a virtual electronic tongue based on electrochemical impedance spectroscopy (EIS) and artificial neural networks (ANNs) as a powerful strategy for detecting and quantifying gasoline adulteration. The impedance profiles revealed that single-adulterant systems follow a clear trend of charge transfer resistance, increasing in the order *n*-hexane < mineral turpentine < toluene, which reflects their molecular interactions with the glassy carbon surface. In contrast, binary and ternary mixtures exhibited nonmonotonic behavior, indicating that intermolecular interactions and competition among species significantly influence the electrochemical response beyond simple additive effects. Complementary FTIR analyses provided spectroscopic support for these observations. Focusing the ANN input on the semicircle region of the Nyquist plots led to markedly improved model performance, enabling no misclassifications in the test set for adulterant type in the test set and enhanced regression capability for individual adulterants, including in mixed systems. These findings highlight that the semicircle region concentrates the most informative features for data-driven analysis and that EIS–ANN integration enables a reagent-free, rapid, and reliable approach to monitor fuel quality. Beyond laboratory conditions, the methodology holds promise for portable diagnostic tools aimed at mitigating the economic and environmental impacts of fuel adulteration.

## Supplementary Material



## Notes

Experimental details; composition of nonadulterated and adulterated gasoline samples (Table S1); parameters used for ANN classification and regression models; cyclic voltammogram used for electrode validation (Figure S1); EIS data for gasoline samples adulterated with ternary and binary mixtures (Figures S2, S5, S6, and S8); FTIR spectra of pure compounds and gasoline samples (Figures S3, S4, and S7); and FTIR band assignments (Table S2) (PDF)
