# Supplementary material for: Virtual Electronic Tongue Combining Electrochemical Impedance Spectroscopy and the Artificial Neural Network for Accurate Identification of Noncompliant Gasoline
Source: ACS Omega. 2026 Feb 27;11(9):14381–91. doi: 10.1021/acsomega.5c08979 (PMC12980438; doi:10.1021/acsomega.5c08979)
Supplement: Supplementary file 1 [file ao5c08979_si_001.pdf]

SUPPLEMENTARY MATERIAL

A Virtual Electronic Tongue Combining Electrochemical Impedance Spectroscopy and Artificial Neural Network for Accurate Identification of Non-Compliant Gasoline

Bianca de Paula Cola[1], André Guimarães de Oliveira[2], Ana Maria Rocco[2], Maiara Oliveira Salles[1*]

[1]Instituto de Química, Universidade Federal do Rio de Janeiro, Brasil

[2] Conductive Materials and Energy Group, Chemical and Biochemical Engineering Processes, School of Chemistry, UFRJ, Universidade Federal do Rio de Janeiro, Centro de Tecnologia, Bloco E, Rio de Janeiro, CEP: 21941-909, RJ, Brazil

Corresponding Author: maiara@iq.ufrj.br

1. EXPERIMENTAL SECTION

Table S1 – Composition of the gasoline samples not adulterated and adulterated with toluene, turpentine and n-hexane.

Number of the sample	Sample code	% of toluene	% of turpentine	% of n-hexane
**1**	0TO_0TU_10HX	0.0	0.0	10.0
**2**	0TO_3TU_7HX	0.0	3.0	7.0
**3**	0TO_3TU_10HX	0.0	3.0	10.0
**4**	0TO_7TU_3HX	0.0	7.0	3.0
**5**	0TO_7TU_7HX	0.0	7.0	7.0
**6**	0TO_7TU_10HX	0.0	7.0	10.0
**7**	0TO_10TU_0HX	0.0	10.0	0.0
**8**	0TO_10TU_3HX	0.0	10.0	3.0
**9**	0TO_10TU_7HX	0.0	10.0	7.0
**10**	0TO_10TU_10HX	0.0	10.0	10.0
**11**	3TO_0TU_7HX	3.0	0.0	7.0
**12**	3TO_0TU_10HX	3.0	0.0	10.0
**13**	3TO_3TU_3HX	3.0	3.0	3.0
**14**	3TO_3TU_7HX	3.0	3.0	7.0
**15**	3TO_3TU_10HX	3.0	3.0	10.0
**16**	3TO_7TU_0HX	3.0	7.0	0.0
**17**	3TO_7TU_3HX	3.0	7.0	3.0
**18**	3TO_7TU_7HX	3.0	7.0	7.0
**19**	3TO_7TU_10HX	3.0	7.0	10.0
**20**	3TO_10TU_0HX	3.0	10.0	0.0
**21**	3TO_10TU_3HX	3.0	10.0	3.0
**22**	3TO_10TU_7HX	3.0	10.0	7.0
**23**	3TO_10TU_10HX	3.0	10.0	10.0
**24**	7TO_0TU_3HX	7.0	0.0	3.0
**25**	7TO_0TU_7HX	7.0	0.0	7.0
**26**	7TO_0TU_10HX	7.0	0.0	10.0
**27**	7TO_3TU_0HX	7.0	3.0	0.0
**28**	7TO_3TU_3HX	7.0	3.0	3.0
**29**	7TO_3TU_7HX	7.0	3.0	7.0
**30**	7TO_3TU_10HX	7.0	3.0	10.0
**31**	7TO_7TU_0HX	7.0	7.0	0.0
**32**	7TO_7TU_3HX	7.0	7.0	3.0
**33**	7TO_7TU_7HX	7.0	7.0	7.0
**34**	7TO_7TU_10HX	7.0	7.0	10.0
**35**	7TO_10TU_0HX	7.0	10.0	0.0
**36**	7TO_10TU_3HX	7.0	10.0	3.0
**37**	7TO_10TU_7HX	7.0	10.0	7.0
**38**	7TO_10TU_10HX	7.0	10.0	10.0

**39**	10TO_0TU_OHX	10.0	0.0	0.0
**40**	10TO_0TU_3HX	10.0	0.0	3.0
**41**	10TO_0TU_7HX	10.0	0.0	7.0
**42**	10TO_0TU_10HX	10.0	0.0	10.0
**43**	10TO_3TU_0HX	10.0	3.0	0.0
**44**	10TO_3TU_3HX	10.0	3.0	3.0
**45**	10TO_3TU_7HX	10.0	3.0	7.0
**46**	10TO_3TU_10HX	10.0	3.0	10.0
**47**	10TO_7TU_0HX	10.0	7.0	0.0
**48**	10TO_7TU_3HX	10.0	7.0	3.0
**49**	10TO_7TU_7HX	10.0	7.0	7.0
**50**	10TO_7TU_10HX	10.0	7.0	10.0
**51**	10TO_10TU_0HX	10.0	10.0	0.0
**52**	10TO_10TU_3HX	10.0	10.0	3.0
**53**	10TO_10TU_7HX	10.0	10.0	7.0
**54**	10TO_10TU_10HX	10.0	10.0	10.0
**Non-adulterated gasoline 1**		0.0	0.0	0.0
**Non-adulterated gasoline 2**		0.0	0.0	0.0
**Non-adulterated gasoline 3**		0.0	0.0	0.0
**Non-adulterated gasoline 4**		0.0	0.0	0.0
**Non-adulterated gasoline 5**		0.0	0.0	0.0

1.1 Parameters used for evaluating ANN models

1.1.1 Classification model

For the classification model, the following parameters were used (for all metrics, values closer to 1 indicate better performance):

- **AUC** (Area Under the ROC Curve): Quantifies the probability that the classifier ranks a randomly chosen positive instance higher than a randomly chosen negative one. It is obtained by calculating the area under the Receiver Operating Characteristic (ROC) curve, which plots the True Positive Rate (Recall) against the False Positive Rate for all possible decision thresholds. An AUC of 0.5 indicates random performance, while 1.0 indicates perfect discrimination.

- **CA** (Classification Accuracy): Proportion of correct predictions over the total number of predictions.

$$CA\ (accuracy) = \frac{TP + TN}{TP + TN + FP + FN} \qquad Equation\ 1$$

- **F1** (F1-Score): Harmonic mean of Precision and Recall, useful when there is class imbalance.

$$F1 = 2 * \frac{Precision * Recall}{Precision + Recall} \qquad Equation\ 2$$

- **Precision**: Proportion of correctly predicted positive instances among all predicted positives.

$$Precision = \frac{TP}{TP + FP} \qquad Equation\ 3$$

- **RECALL**: Proportion of actual positive instances correctly identified by the model (Equation 4).

$$Recall = \frac{TP}{TP + FN} \qquad Equation\ 4$$

Where: TP: true positive, TN: true negative, FP: false positive, FN: false negative

1.1.2 Regression models

For regression metrics (MSE, RMSE, MAE), lower values indicate better performance. For R^2, values closer to 1 indicate better performance:

- **MSE** (Mean Squared Error): Average of the squared differences between predicted and actual values, penalizing larger errors more strongly.

$$MSE = \frac{1}{n}\sum_{i=1}^{n}(y_i - \hat{y}_i)^2 \qquad Equation\ 5$$

- **RMSE** (Root Mean Squared Error): Square root of the MSE, keeping the error in the same units as the target variable.

$$RMSE = \sqrt{MSE} \qquad Equation\ 6$$

- **MAE** (Mean Absolute Error): Average of the absolute differences between predicted and actual values, providing a direct interpretation of the average magnitude of the error.

$$MAE = \frac{1}{n}\sum_{i=1}^{n}|y_i - \hat{y}_i| \qquad Equation\ 7$$

- **R^2** (Coefficient of Determination): Proportion of the variance in the dependent variable explained by the model.

Where: n is the total number of observations; y_i is the actual (observed) value for the *i*-th sample; and $\hat{y}_i$ is the predicted value for the *i*-th sample.

2. RESULTS AND DISCUSSION

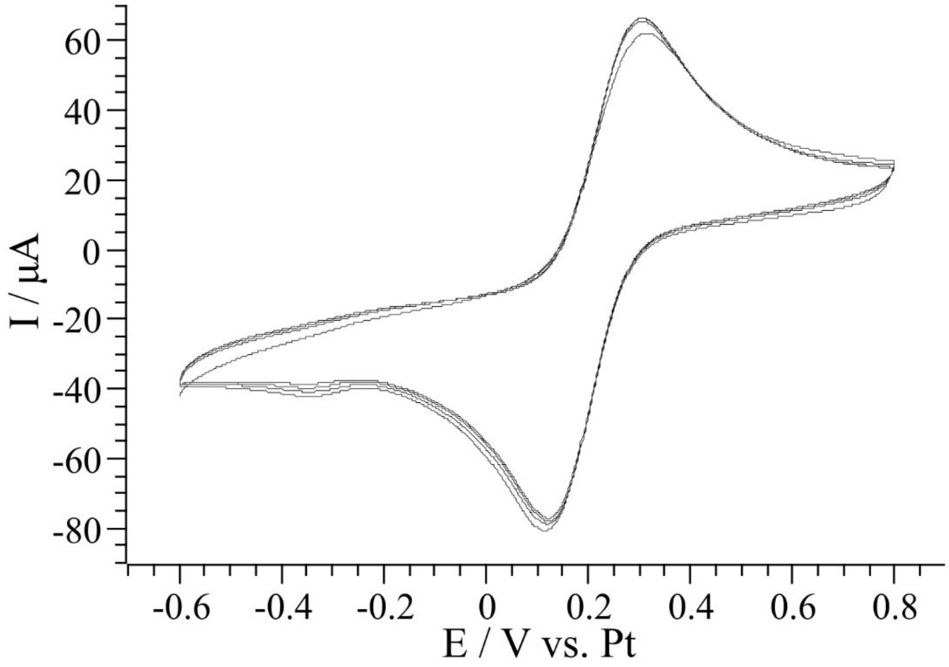

Figure S1 – Cyclic voltammogram obtained with a glassy carbon as working electrode and two platinum wires used as reference and counter electrode in a 0.01 mol L^{-1} $K_3[Fe(CN)_6]$ and 0.1 mol L^{-1} KCl solution. Scan rate: 25 mV s^{-1}.

2.1 Gasoline adulterated with ternary adulterant mixtures

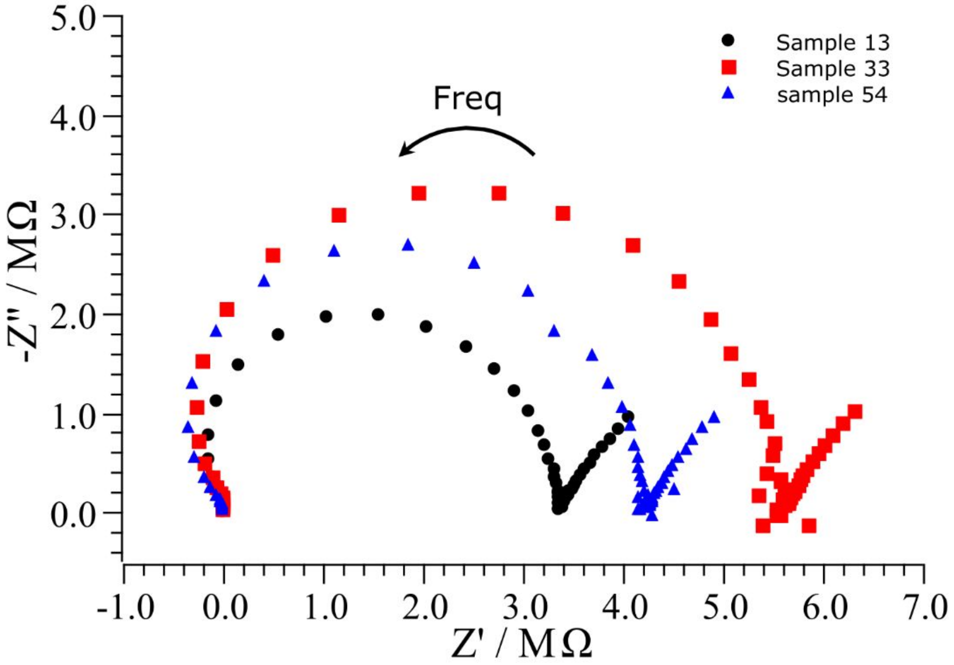

Figure S2 – EIS analysis of gasoline samples adulterated: 3TO_3TU_3HX (sample 13 – black circle); 7TO_7TU_7HX (sample 33 – red square); 10TO_10TU_10HX (sample 54 – blue triangle). Glassy carbon as working electrode, two Pt wires as reference and counter electrodes. Measurements were carried out

over a frequency range from 10^5 Hz to 0.1 Hz, using a 10 frequency per decade distribution and a sinusoidal perturbation of 0.06 VRMS, relative to the open-circuit potential (OCP).

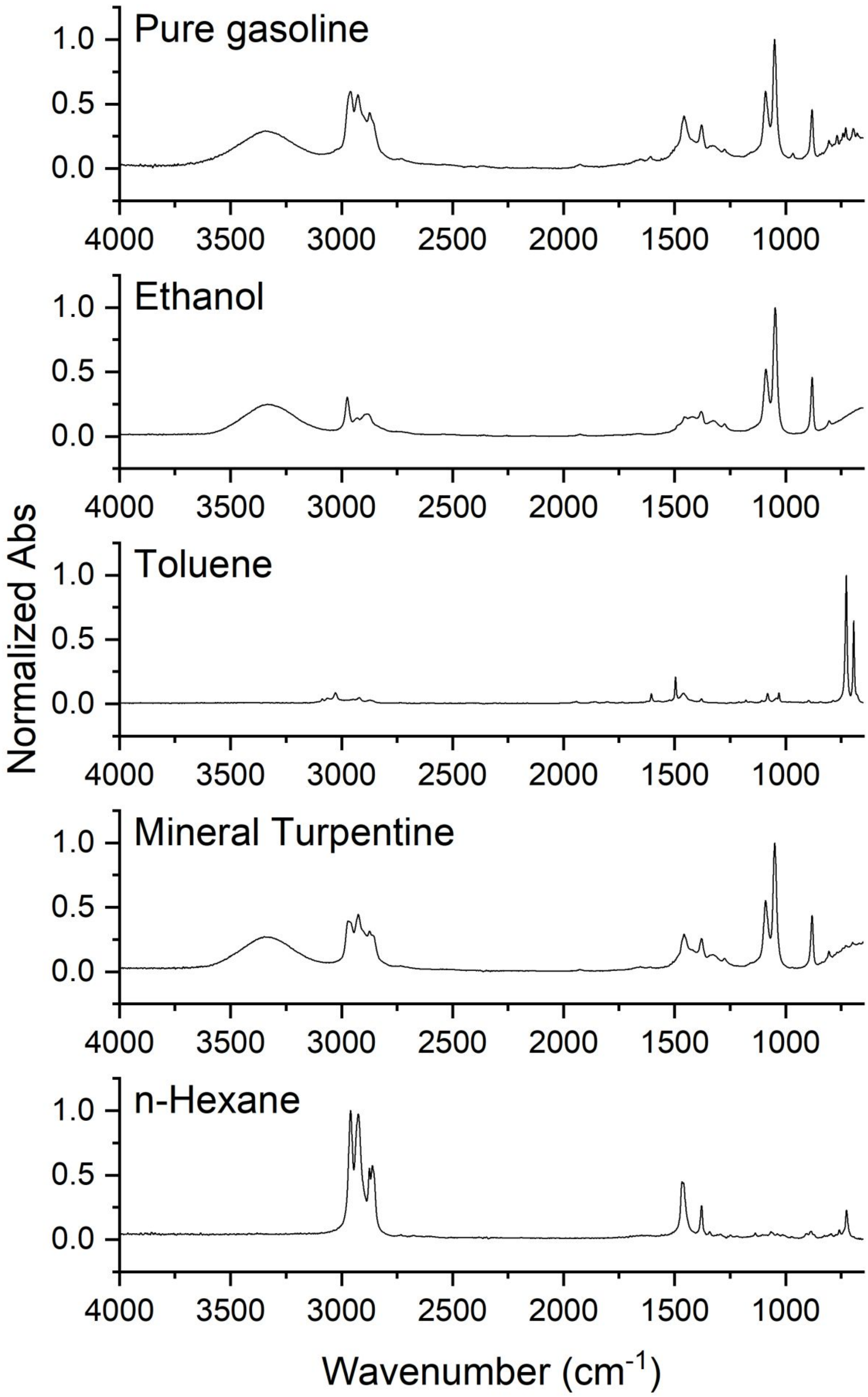

Figure S3: (a) Normalized FTIR spectra of non-adulterated gasoline, ethanol, toluene, mineral turpentine and n-hexane.

Table S2: FTIR-Based molecular vibration assignments for the spectra shown in Figure S2.

**Compound**	**Wavelength (cm^{-1})**	**Peak Attribution**	**Functional Group or Molecular Vibration**
**Gasoline**	2960–2870	ν_a(C–H) stretching (alkyl groups)	Asymmetric and symmetric C–H stretching in methylene (–CH_2–) and methyl (–CH_3) groups in alkanes.
	1450–1370	δ(C–H) bending (methylene and methyl groups)	C–H bending in CH_2 and CH_3 groups of alkanes.
	875	δ(C–H) out-of-plane bending (aromatic)	Out-of-plane C–H bending in monocyclic aromatic hydrocarbons (e.g., toluene).
	720	δ(C–H) out-of-plane bending (aromatic)	Out-of-plane C–H bending vibrations in monosubstituted aromatic rings (e.g., toluene).
	1600	ν(C=C) stretching (aromatic)	C=C stretching in aromatic rings (e.g., benzene).
	3000–3100	ν_a(C–H) stretching (aromatic)	Aromatic C–H stretching (in toluene, xylene).
**Ethanol**	3400–3200	ν_a(O–H)	O–H stretching (hydroxyl group in ethanol)
	2960–2870	ν_a(C–H)	C–H stretching (alkyl groups in ethanol)
	1460	δ(C–H)	C–H bending (methylene and methyl groups in ethanol)
	1380	δ(C–H)	C–H bending (methylene groups in ethanol)
	1050	ν(C–O)	C–O stretching (alcoholic C–O bond in ethanol)
	1150–1100	δ(C–C–O)	C–C–O bending (ether-like structures, or C–O–C linkages)

**Toluene**	3030–3080	ν_a(C–H) stretching (aromatic)	Aromatic C–H stretching in toluene.
	1600	ν(C=C) stretching (aromatic)	C=C stretching in the aromatic ring (toluene).
	695–705	δ(C–H) out-of-plane bending (aromatic)	Out-of-plane C–H bending in monosubstituted aromatics (toluene).
	770	δ(C–H) out-of-plane bending (aromatic)	Out-of-plane C–H bending in monosubstituted aromatics (toluene).
**Mineral Turpentine**	2960–2870	ν_a(C–H) stretching (alkyl groups)	Asymmetric and symmetric C–H stretching in methylene (–CH_2–) and methyl (–CH_3) groups in alkanes.
	1450–1370	δ(C–H) bending (methylene and methyl groups)	C–H bending in CH_2 and CH_3 groups of alkanes.
	1080	ν_a(C–O) vibrations	C–O stretching in ether or alcohol groups.
	1040	ν_a(C–C) stretching (alkyl groups)	C–C stretching in alkyl groups (e.g., alkanes, cycloalkanes).
	725–690	δ(C–H) out-of-plane bending (aromatic)	Out-of-plane C–H bending in monosubstituted aromatic rings (e.g., toluene, xylene).
	1600	ν(C=C) stretching (aromatic)	C=C stretching in aromatic rings (mainly in toluene, xylene).
	2900	ν_a(C–H) stretching (aromatic)	Aromatic C–H stretching in toluene and xylene.
	676–678	δ(C–H) out-of-plane bending (aromatic)	Out-of-plane C–H bending in monosubstituted aromatics (e.g., toluene).
**Hexane**	2960–2870	ν_a(C–H) stretching (alkyl groups)	Asymmetric and symmetric C–H stretching in methylene (–CH_2–) and methyl (–CH_3) groups in alkanes.

1460	δ(C–H) bending (methylene and methyl groups)	C–H bending in CH_2 and CH_3 groups of alkanes.
720	δ(C–H) out-of-plane bending (alkyl groups)	Out-of-plane C–H bending in monosubstituted alkyl groups (hexane).
1370	δ(C–H) bending (alkyl groups)	C–H bending in CH_2 and CH_3 groups of alkanes.

Electrochemical data shown in Figure S1 indicate that distinct molecular interactions may develop at specific concentration intervals. To explore this possibility, FTIR measurements were performed. Figure S2 shows the FTIR spectra of non-adulterated gasoline and of the ternary mixtures 33 (7TO–7TU–7HX) and 54 (10TO–10TU–10HX).

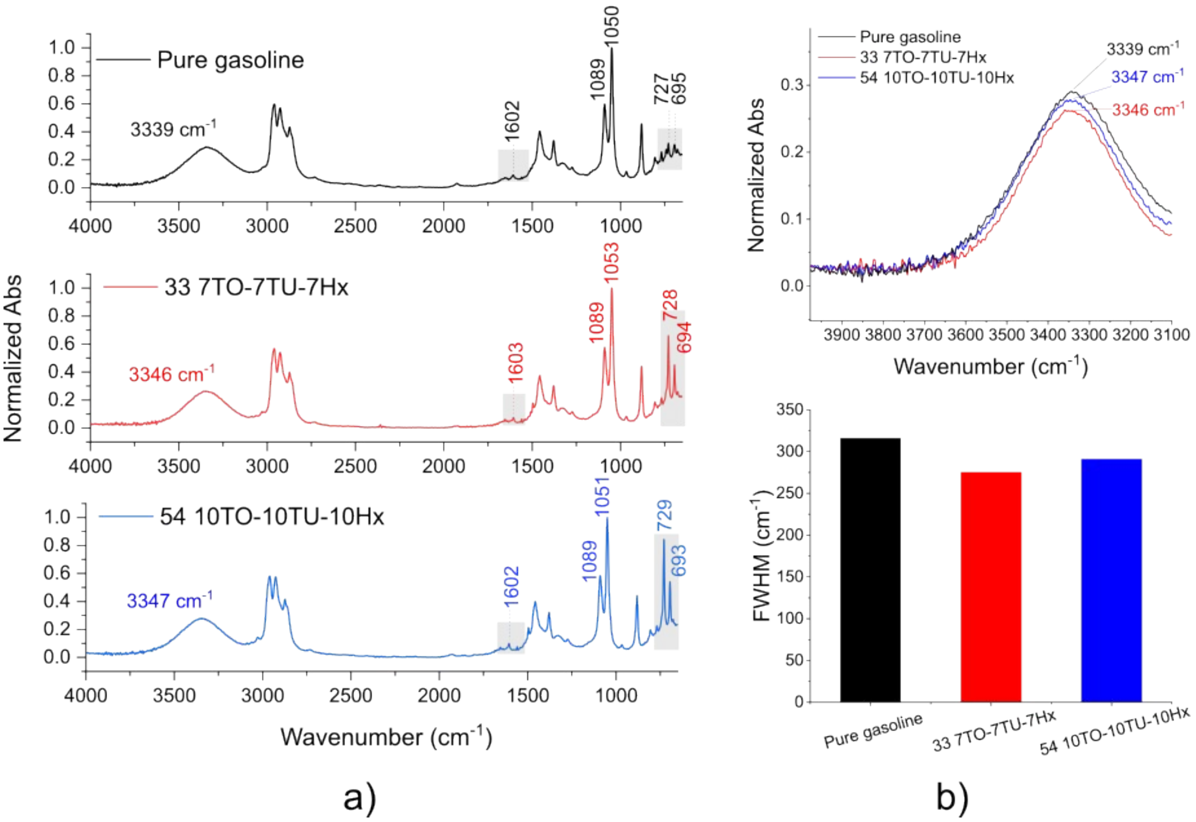

Figure S4 – (a) Normalized FTIR spectra of non-adulterated gasoline (black), and adulterated samples 33 7TO–7TU–7Hx (red) and 54 10TO–10TU–10Hx (blue). (b) In detail, characteristic bands associated with ethanol combination/overtone transitions appear near 3347–3339 cm⁻¹ and calculated FWHM for ethanol band for each sample.

2.2 Gasoline adulterated with binary adulterant mixtures

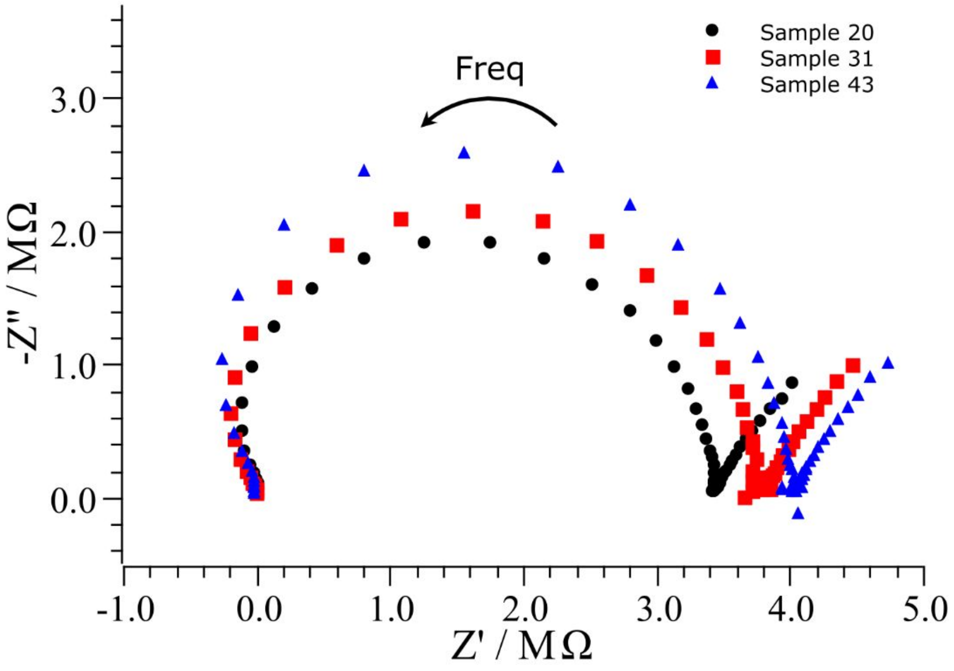

Figure S5 – EIS analysis of gasoline samples adulterated with 3TO_10TU_0HX (sample 20 – black circle); 7TO_7TU_0HX (sample 31 – red square) and 10TO_3TU_0HX (sample 43 – blue triangle). Glassy carbon as working electrode, two Pt wires as reference and counter electrodes. Measurements were carried out over a frequency range from 10^5 Hz to 0.1 Hz, using a 10 frequency per decade distribution and a sinusoidal perturbation of 0.06 VRMS, relative to the open-circuit potential (OCP).

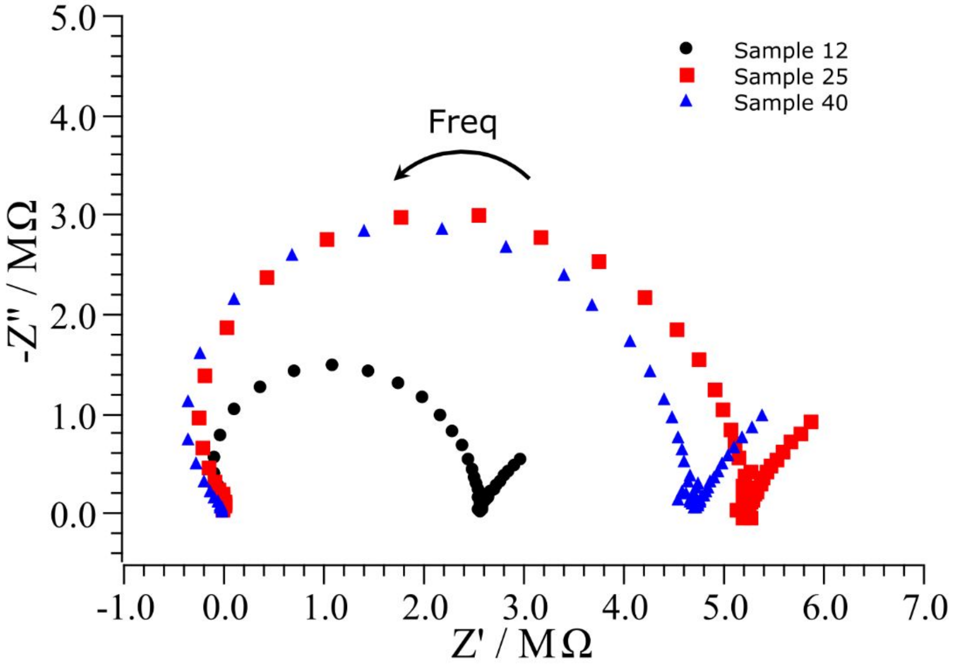

Figure S6 – EIS analysis of gasoline samples adulterated with 3TO_0TU_10HX (sample 12 – black circle); 7TO_0TU_7HX (sample 25 – red square) and 10TO_0TU_3HX (sample 40 – blue triangle). Glassy carbon as working electrode, two Pt wires as reference and counter electrodes. Measurements were carried out over a frequency range from 10^5 Hz to 0.1 Hz, using a 10 frequency per decade distribution and a sinusoidal perturbation of 0.06 VRMS, relative to the open-circuit potential (OCP).

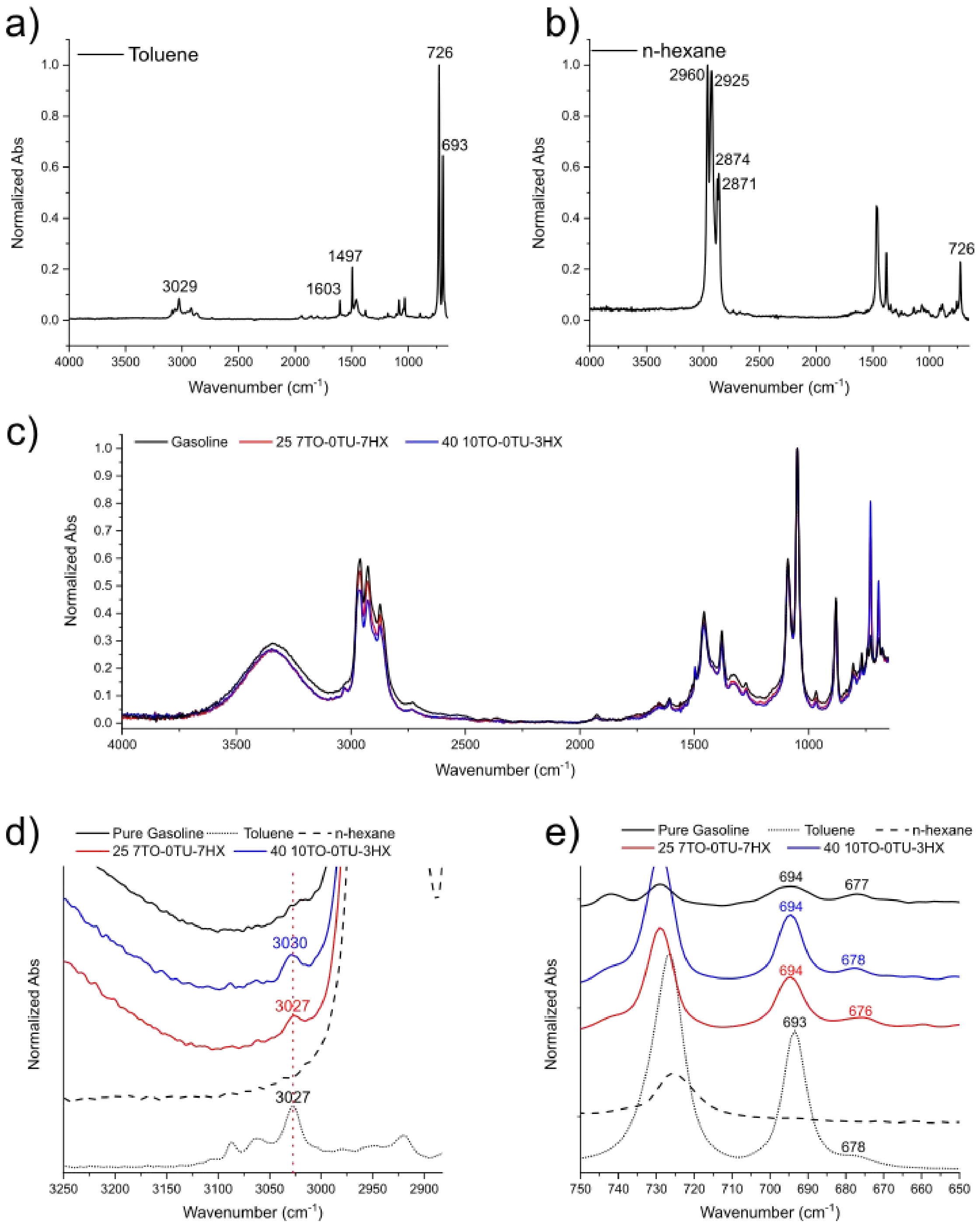

Figure S7 – (a) Normalized FTIR spectra of Toluene. (b) Normalized FTIR spectra of n-hexane. (c) Normalized FTIR spectra of non-adulterated gasoline (black), and adulterated samples 25 7TO–0TU–7Hx (red) and 40 10TO–0TU–3Hx (blue). (d) Amplified region from 3250-2900 cm^{-1} . (e) Amplified region from 750-650 cm^{-1}.

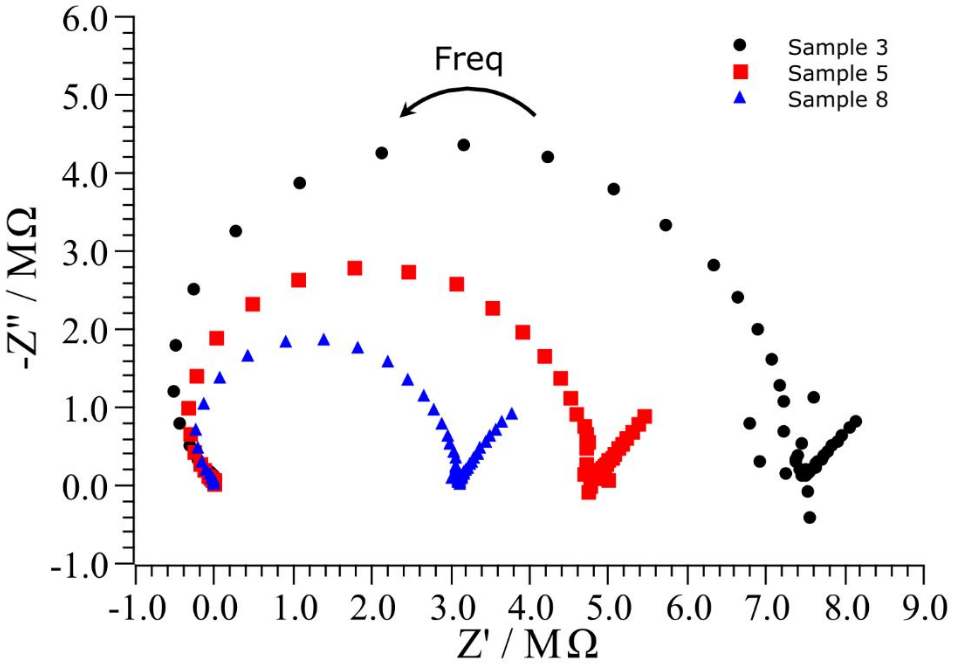

Figure S8 – EIS analysis of gasoline samples adulterated with 0TO_3TU_10HX (sample 3 – black circle); 0TO_7TU_7HX (sample 5 – red square) and 0TO_10TU_3HX (sample 8 – blue triangle). Glassy carbon as working electrode, two Pt wires as reference and counter electrodes. Measurements were carried out over a frequency range from 10^5 Hz to 0.1 Hz, using a 10 frequency per decade distribution and a sinusoidal perturbation of 0.06 VRMS, relative to the open-circuit potential (OCP).
